# Body weight perception among Sri Lankan cardiac patients

**DOI:** 10.1186/s40608-016-0113-5

**Published:** 2016-07-04

**Authors:** Ranil Jayawardena, Pavani Punchihewa, Ishara Ranathunga, Niroshan Lokunarangoda, Anidu Keerthi Pathirana, Wijeyasingam Samuel Santharaj

**Affiliations:** Department of Physiology, Faculty of Medicine, University of Colombo, Colombo, Sri Lanka; Institute of Health and Biomedical Innovation, Queensland University of Technology, Brisbane, Queensland Australia; Institute of Cardiology, National Hospital of Sri Lanka, Colombo, Sri Lanka; Department of Medicine, Faculty of Medicine and Allied Sciences, University of Rajarata, Mihintale, Sri Lanka

**Keywords:** Weight perception, Cardiac, Body mass index, Sri Lankan, Overweight, obese

## Abstract

**Background:**

Misperception of body weight by individuals is a known occurrence. However, it is a potential target for implementing obesity reduction interventions in patients with cardiovascular and metabolic diseases. The aim of this study was to describe the association between self-perception of body weight and objectively measured body mass index (BMI) among cardiac patients in a specialist cardiology institution in Sri Lanka.

**Method:**

During the study period, 322 (61 %) males and 204 (39 %) females were recruited from consecutive admissions to the Institute of Cardiology, National Hospital, Colombo, Sri Lanka. An interviewer-administered questionnaire was used to assess demographic characteristics, medical records and body weight perception. Weight, height and waist circumference (WC) were measured and Asian anthropometric cut-off points for BMI and WC were applied.

**Results:**

The mean BMI of the study population was 23.61 kg/m^2^. Body size misperception was seen in a significant proportion of the cohort. 85.2 % of overweight patients reported themselves to be of ‘normal weight’ or even ‘underweight’. Moreover, 36 % of obese patients misperceived body weight as being of ‘normal weight’ while 10.9 % considered themselves to be ‘underweight’. 61.9 % of males and 68.8 % of females with central obesity reported themselves to be ‘underweight’ or ‘normal weight’. Among a subgroup with co-morbid metabolic diseases, significant under-perception of body size was seen.

**Conclusions:**

Significant body size misperceptions were noted in this group of cardiac patients. The disparity of perception was seen increasingly with increasing BMI. More than two thirds of overweight and more than half of obese patients believed themselves to have normal or less than normal weight.

**Electronic supplementary material:**

The online version of this article (doi:10.1186/s40608-016-0113-5) contains supplementary material, which is available to authorized users.

## Background

Obesity is currently considered to be a global epidemic [1]. The World Health Organisation (WHO) estimates that the worldwide prevalence of obesity has more than doubled between the years of 1980 and 2014 [2]. In the latest estimate in 2014, 1.9 billion adults were expected to be overweight, among whom over 600 million were projected to be obese [2]. Obesity is a major determinant of cardiovascular disease and such associations between them have been widely studied. Profound links have been established between hypertension, dyslipidaemia and obesity [3]. Obesity is known to be a major modifiable risk factor for coronary artery disease [4]. According to the WHO cut-off values for Asians, 25.2 %, 9.2 % and 26.2% of Sri Lankan adult population are in the overweight, obese and centrally obese categories respectively [5]. In Sri Lanka, the level of obesity has increased 3 fold duringthe last two decades. (5). The nation suffers notably from obesity associated metabolic diseases such as diabetes [6], hypertension [6], and metabolic syndrome [5] on an epidemic level.

In order to implement weight reduction strategies with the ultimate goal of reducing adverse health outcomes, it is important that the patients truly perceive their excess body weight as a modifiable risk factor. It is known that obese individuals with misperception of the body size are less aware of their lifetime risks associated with the disease and have overly optimistic beliefs regarding their health [7]. This issue is further complicated by lower health seeking behaviour and less active participation in health promoting lifestyle interventions[7].The implication of these is the invariable impact on success or otherwise of preventive measures. Body size misperception has been identified as a challenge and a potential target for implementing obesity reduction interventions [7]. However, such misperceptions are likely to have detrimental effects on measures to be taken towards primary prevention of the above listed metabolic and cardiovascular disorders.

There are several reports from different healthy populations, where significant misperceptions of weight have been recognized [8–10]. The changes in public perception of weight and improvement in public awareness of the significance of this parameter over the years has also been studied. These have concluded that despite the increasing awareness programmes, public perceptions have not improved satisfactorily [11]. Association between body weight perception and metabolic disorders has also been studied. Significant discrepancies have been found between self-perceived and objectively measured body weight status in type 2 Diabetes patients [12]. Clinical outcomes with regard to blood pressure and blood sugar control in diabetic patients are also known to be affected by this distortion of body weight perception [12]. Similar studies have revealed significant weight misperceptions in hypertensive patients as well [13].

The importance of studying this area of interest in the subgroup of cardiac patients is because their perception may differ from that of the general population. As cardiovascular disease is known to be prevalent among patients with diabetes [14] and dyslipidaemia, which in turn have established associations with obesity [15], the perception of body weight in relation to these comorbidities in cardiac patients should also be studied.

With obesity being a contributory factor for development and progression of chronic diseases including cardiovascular disease, weight reduction has proven to be effective in prevention and treatment [16]. Cardiac patients may be expected to have a higher level of awareness and involvement with body weight control. Accurate weight perception is a motivating factor for weight reduction and is identified to correlate with weight control practices rather than the actual body weight [9].

Accurate body weight perception can be influenced by several socio-demographic factors including age, gender, level of education, occupation and past medical history [13]. Having medical disorders such as diabetes and dyslipidaemia increases the accuracy of own body weight perception [13]. The current study aims to describe weight perception in the context of general cardiac patients and patients with co-morbid metabolic diseases.

## Method

The cardiology unit of a tertiary care hospital was selected as the study setting and data was collected from consecutive admissions between March 2012 and July 2012. From these, patients with certain circumstances precluding accurate anthropometric measurements, those who were unable to respond to questions administered and those who were admitted for a specific investigation or intervention were excluded. Furthermore pregnant or lactating women, patients with acute oleander poisoning were not recruited. Details of another different aspect of the same the study have been reported elsewhere [17].

A custom designed, interviewer-administered questionnaire was used. This included socio-demographic data, medical history of current illness, co-morbidities, subjective assessment of the body weight and objective assessment through standard anthropometric measurements. Data was collected by a trained medical officer. Only patients giving informed written consent were included. This study protocol was approved by the Ethical Review Committee of National Hospital of Sri Lanka.

Individual body weight perception was inquired into and it was described in terms of “underweight”, “right weight”, “overweight” and “very overweight”. Body weight was measured to the nearest 100 grams using an electronic scale (Seca 815, Seca GmbH. Co. kg, Germany) and height was measured to the nearest millilitre using a stadiometer (Seca 217, Seca GmbH. Co. kg, Germany). Body Mass Index (BMI) was calculated as weight in kilograms divided by height squared in meters. Waist circumference (WC) was measured midway between the iliac crest and lower rib margin at the end of normal expiration using a standard measuring tape to the nearest 0.1 cm.

Patients were classified into four groups according to their measured BMI values, and cut-off values from Sri Lankan guidelines were used for this categorisation; underweight <18.5 kg/m^2^, normal weight 18.5-22.9 kg/m^2^, overweight 23–25 kg/m^2^, obese >25 kg/m^2^ [18]. WC values exceeding 90 cm in males and 80 cm in females were was used as the cut-off values for central obesity [18]. Patients with obesity associated diseases such as diabetes mellitus, hypertension and dyslipidaemia were defined as co-morbid diseases.

Data entry and analysis was done using SPSS Version 20.0 statistical package.

## Results

Total number of patients enrolled were 526 (response rate 100 %), out of which 322 (61 %) were males. Mean age was 58.5 years (±12.0). Majority of the population were Sinhalese (*n* = 438, 83 %), whereas Muslims (*n* = 43, 8 %), Sri Lankan Tamils (*n* = 34, 7 %), Indian Tamils (*n* = 4, 1 %) and other ethnic groups (*n* = 7, 1 %) constituted the remainder of the group. About half of the population (*n* = 296) had secondary or higher education and 10.5% (*n* = 55) had no formal education.From these patients, the largest proportion (*n* = 275, 52 %) presented with Acute Coronary Syndrome. Arrhythmias accounted for 13 % (*n* = 67), heart failure and cardiomyopathies accounted for 11 % (*n* = 59), representing the other common diagnostic category. Socio-demographic characteristics of the study population are summarized in Table 1.

**Table 1:** Socio-demographic and clinical characteristics of patients in the population

Patient characteristic	Male		Female		Total	
	*n* = 322		*n* = 204		*n* = 526	
	number	%	number	%	number	%
*Ethnicity*						
Sinhala	266	82.6	172	84.3	438	83.3
Muslim	24	7.5	19	9.3	43	8.2
Indian Tamil	4	1.2	0	0	4	0.8
Sri Lankan Tamil	22	6.8	12	5.9	34	6.5
*Education Level*						
No formal education	25	7.8	30	14.7	55	10.5
Primary	96	29.8	79	38.7	175	33.3
Secondary	186	57.8	87	42.6	273	51.9
Tertiary	15	4.7	8	3.9	23	4.4
*Diagnosis*						
IHD	87	42.6	275	52.3	188	58.4
Arrhythmia	31	15.2	67	12.7	36	11.2
Cardiomyopathy/HF	9	4.4	16	3	7	2.2
Valvular heart disease	26	12.7	59	11.2	33	10.2
Infections	8	3.9	22	4.2	14	4.3
Pericardial diseases	4	2	7	1.3	3	0.9
Iry/IIry lung disease	11	5.4	15	2.9	4	1.2
Miscellaneous	28	13.8	65	12.3	37	11.5

The mean value with the Standard Deviation (SD) of the BMI of the study population was 23.6 (±4.2)kg/m^2^. Over one third of the subjects population (35 %, *n* = 183) had normal weight and an almost equally high percentage (*n* = 175, 33 %) were obese as well. 22 % (*n* = 115) were overweight and 10 % (*n* = 52) were underweight. More than half of the study sample (58 %, *n* = 306) had central obesity which constituted 43% (*n* = 139) males and 82 % (*n* = 167) females.

Body weight perception varied with the objectively measured BMI category and between males and females (Table 2). More than a two third proportion of patients with BMI in the underweight category (*n* = 52, 71.2 %) accurately recognized themselves to be underweight. Nearly half of the patients with normal BMI, considered themselves to be ‘underweight’. A majority of patients who were overweight (85.2 %; M: 80.5 %, F: 93.2 %) reported themselves as being ‘normal weight’ or even ‘underweight’. 53 % of obese patients considered themselves to be ‘overweight’ or ‘obese’. However, only 15 % correctly identified the weight category as being ‘very overweight’. More than one third of obese patients misperceived their body weight as being of ‘normal weight’. Moreover, 11 % considered themselves to be ‘underweight’. Overall with the increase in the BMI, lesser percentages of patients correctly perceived their body weight.

**Table 2:** Actual BMI vs Weight perception category

BMI categories (n)		Weight perception		
	“under weight”	“right weight”	“over weight”	“very over weight”
Underweight (<18.5 kg/m2)				
Total(52)	71.2	26.9	1.9	0
Male(25)	60	40	0	0
Female(27)	81.5	14.8	3.7	0
Normal weight (18.5-22.9 kg/m2)				
Total(183)	50.8	44.8	4.4	0
Male(129)	49.6	45	5.4	0
Female(54)	53.7	44.4	1.9	0
Overweight (23–24.9 kg/m2)				
Total(115)	22.6	62.6	14.8	0
Male(72)	19.4	61.1	19.4	0
Female(43)	27.30	65.9	6.8	0
Obese (>25 kg/m2)				
Total(175)	10.9	36	38.3	14.9
Male(96 )	11.5	41.7	33.3	13.5
Female(79)	10.1	29.1	44.3	16.5

A total of 62 % males and 69 % females with central obesity, reported themselves to be ‘underweight’ or ‘normal weight’. Those who perceived their weight to be ‘overweight’ or ‘very overweight’ constituted about one third of the total number of patients with central obesity (Table 3).

**Table 3:** Weight perception changes with Central obesity^a^

	Weight perception category			
	"Underweight" (%)	"Right weight" (%)	"Overweight" (%)	"Very overweight" (%)
Central obesity^a^ (*n*)				
Males				
Absent (183)	45.9	47	7.1	0
Present (139)	14.4	47.5	28.8	9.4
Females				
Absent (37)	78.4	18.9	2.7	0
Present(167)	25.1	43.7	23.4	7.8

^a^Central obesity = WC > 90 cm in males, >80 cm in females

Table 4 summarizes body weight perception variations in cardiac patients with co-morbid metabolic diseases.A total of 85 % overweight patients and 50 % obese patients in this sub-category identified their weight as ‘underweight’ or ‘normal weight’.

**Table 4:** Weight perception changes in cardiac patients with co-morbid metabolic diseases

BMI categories	Weight perception			
	"under weight" (%)	"right weight" (%)	"over weight" (%)	"very over weight" (%)
Underweight (<18.5 kg/m2)	70.6	23.5	5.9	0
Normal weight (18.5-22.9 kg/m2)	54.4	41.1	4.4	0
Overweight (23–24.9 kg/m2)	21.8	62.8	15.4	0
Obese (>25 kg/m2)	10.8	39.2	35.4	14.6

## Discussion

There is limited evidence in the world literature on weight perception variability in cardiac patients. The current study sample showed a representative distribution of socio-economic characteristics of a Sri Lankan population.The mean age of the study sample recruited is 58.5 years and this reflects the fact that cardiac patients are usually older. However, this study cohort is likely to be representative of Sri Lankan cardiac patients. Furthermore, with a large subject population of varying cardiovascular diagnoses, it provides an accurate representation of cardiac patients. Having been conducted in a group of patients who have direct implications of obesity on the causation and progression of their cardiovascular disease [3], and related co-morbidities, this study provides information which is important from the perspective of managing these types of patients.

A majority of the study population (55 %, *n* = 290) had higher than normal BMI values. This is perhaps to be expected considering their possible disease associations. Similarly, a high percentage (58 %, *n* = 306) had central obesity. This is in contrast to the 34 % of abdominally obese subject percentage noted in a Sri Lankan general population [19]. But in both studies, the prevalence of central obesity is higher among female participants compared to male counter part.

Body weight perception in this study group showed a clear tendency of under-estimation of their weight by the patients. This is evident by nearly half of the normal weight patients considering themselves to be ‘underweight’, 85 % of overweight reporting their weight as ‘normal’ or even ‘underweight’ and 85 % of obese patients failing to identify that they are ‘very overweight’. Similar weight misperceptions have been elicited in the Sri Lankan general population as well [19]. Yet for all that, the percentages of under-perception are higher in the current study sample. This under-perception is significant as these patients are in fact expected to have a better understanding of the concept of BMI and individual weight categories, with the information being targeted to them through the health care sector and the media in Sri Lanka. Although there are several awareness programmes on obesity and associated cardio-metabolic risks in both community and clinical settings, our findings showed that the expected inputs have perhaps failed to reach them adequately. Furthermore, the results indicate that higher the BMI, lesser are the percentages of patients who could accurately identify their weight category. More seriously, 23 % of those who were overweight and 11 % of overtly obese patients considered themselves to be ‘underweight’. Such misconceptions would not only prevent them from attempting to lose weight but will also put them at a higher risk for further increasing their weight as well.

In a previous study done in the sub-group of diabetic patients, significant distortions in self-perceived body weight have been found. In fact, this increased significantly with the increase in BMI weight status [12]. This is similar to the observations made in cardiac patients in the current study. Only 12 % of overweight and obese patients in a group of hypertensive patients correctly identified that they had higher than normal weight [13]. In the present group, only around 14 % of both overweight and obese patients managed to accurately state their weight status. These similarities observed between these patient groups, in contrast to the general population, could be expected, as the diseases themselves are most of the time inter-related or co-existent and there are similarities in the exposure of patients to health-related information.

Waist circumference is a more accurate indicator of the metabolic risk in South Asian populations [20]. Being a seminal criterion in defining the metabolic syndrome, it has a major impact on the development of metabolic diseases. Thus the patients with abdominal obesity, especially when they have already developed consequent metabolic or cardiovascular disease, should be aware of it. However the results indicate poor perception, with 62 % of abdominally obese males and 69 % of similar females reporting their weight status as ‘normal’ or ‘underweight’. Only one third of them considered themselves to have higher than normal weight. One could expect problems in them in a therapeutic attempt towards motivation towards weight loss in practices. This is in contrast to the Sri Lankan general population where two thirds of abdominally obese people accurately identified themselves to have an increased waist circumference [19].

The known medical complications of obesity include metabolic diseases such as diabetes, hypertension, hyperlipidaemia as well as ischaemic heart disease [3]. The selected sub-group of cardiac patients with co-morbid metabolic diseases showed an equal trend of body weight misperception with almost similar percentages in different self-perceived weight categories. This would lead one to the inevitable conclusion that weight misperception, especially under-perception when overweight, being a significant finding in the general cardiac patients as well as cardiac patients with co-morbid metabolic diseases. This is apparently more marked than misperceptions demonstrated in the general population from large-scale local studies [19]. Furthermore, this is quite important in view of the fact that this is the same patient population which is expected to be more vigilant about weight control. This misperception may be contributed to by several reasons. Unintentional weight loss is a symptom of diabetes. Furthermore, these patients may have already met health care personnel, including dieticians, from whom they may have received advice on diet and physical exercise, and may even have tried to comply with the suggestions to a certain extent. This could possibly have led to an impression formed within them that they have achieved some weight loss. However the negative impact of it is the failure to recognize that an optimal weight status has not been achieved yet. This subsequently results in under-perception of body weight. The results of this study bring out the notion that target populations should be given special attention in addressing weight status and clinicians should try to improve weight perception in these patients in order to enhance weight control practices.

### Limitations

The present study did not specifically test for the understanding of the concept of central obesity in terms of perceived waist circumference. This could have been important to elucidate awareness of patients on this important clinical parameter. However, in a clinical perspective, weight loss practices that the patients are expected to follow would depend on the overall self-estimation of body weight. This study has not dealt with the association of self-perceived body weight with weight control practices in the cardiac patients and the effectiveness of such. Clearly, future studies would be needed in that respect.

Multiple logistic regression models were carried out for under-perception, correct perception and over perception of body weight. However clinically significant statistical associations were not elicited (Additional file 1). Probably, the existing knowledge on obesity and associated risk factors could be an associated factor. Further research is very important to identify the risk factors for the poor body weight perception in cardiac patients.

## Conclusions

Body weight misperception was common in this cardiac patient population as well as the subgroup with co-existent metabolic diseases. Significant under perceptions were observed in higher BMI categories with 85% of overweight patients and about half of the obese patients reporting their weight as ‘normal’ or even ‘underweight’. Similarly, about two thirdsof males and females with central obesity, considered themselves to have normal or less than normal weight. With incremental increase in the BMI, distortion in body weight perception was noted to be worse. More focused health sector approaches with emphasis given to making patients aware of their weight, waist circumference and their associations with disease conditions would help to improve this in the future. This should essentially lead to a better perception in the target patient population than that of the general population.

## Abbreviations

BMI, body mass index; WC, waist circumference

## Additional file

Additional file 1: Multiple logistic regression models for under-perception, correct perception and over perception of body weight. (DOCX 17 kb)

## References

[1] Caballero B (2007). The global epidemic of obesity: an overview. Epidemiol Rev.

[2] World Health Organization. (2015, 2015.03.30). Obesity and overweight. Available: http://www.who.int/mediacentre/factsheets/fs311/en/

[3] Krauss RM, Winston M, Fletcher BJ, Grundy SM (1998). Obesity: impact on cardiovascular disease. Circulation.

[4] Eckel RH, Krauss RM (1998). American Heart Association call to action: obesity as a major risk factor for coronary heart disease. AHA Nutrition Committee. Circulation.

[5] Katulanda P, Jayawardena MA, Sheriff MH, Constantine GR, Matthews DR (2010). Prevalence of overweight and obesity in Sri Lankan adults. Obes Rev.

[6] Wijewardene K, Mohideen MR, Mendis S, Fernando DS, Kulathilaka T, Weerasekara D (2005). Prevalence of hypertension, diabetes and obesity: baseline findings of a population based survey in four provinces in Sri Lanka. Ceylon Med J.

[7] Powell TM, de Lemos JA, Banks K, Ayers CR, Rohatgi A, Khera A (2010). Body size misperception: a novel determinant in the obesity epidemic. Arch Intern Med.

[8] Cheung PC, Ip PL, Lam ST, Bibby H (2007). A study on body weight perception and weight control behaviours among adolescents in Hong Kong. Hong Kong Med J.

[9] Wang Y, Liang H, Chen X (2009). Measured body mass index, body weight perception, dissatisfaction and control practices in urban, low-income African American adolescents. BMC Public Health.

[10] Tiggemann M, Rothblum E (1988). "Gender differences in social consequences of perceived overweight in the United States and Australia,". Sex Roles.

[11] Johnson F, Cooke L, Croker H, Wardle J. Changing perceptions of weight in Great Britain: comparison of two population surveys. BMJ. 2008;337:a494. 10.1136/bmj.a494 PMC2500200 18617488

[12] Mogre V, Abedandi R, Salifu ZS (2014). Distorted self-perceived weight status and underestimation of weight status in diabetes mellitus type 2 patients. PLoS One.

[13] Duncan PR, Howe LD, Manukusa Z, Purdy S. Determinants of obesity and perception of weight in hypertensive patients in rural South Africa. South African Journal of Clinical Nutrition. 2014;27:2.

[14] Sowers JR, Epstein M, Frohlich ED (2001). Diabetes, hypertension, and cardiovascular disease: an update. Hypertension.

[15] Jafar TH, Chaturvedi N, Pappas G (2006). Prevalence of overweight and obesity and their association with hypertension and diabetes mellitus in an Indo-Asian population. CMAJ.

[16] C. J. Lavie, R. V. Milani, and H. O. Ventura, "Obesity and Cardiovascular Disease: Risk Factor, Paradox, and Impact of Weight Loss," Journal of the American College of Cardiology, vol. 53, pp. 1925–1932, 5/26/ 2009. 10.1016/j.jacc.2008.12.068 19460605

[17] Pathirana AK, Lokunarangoda N, Ranathunga I, Santharaj WS, Ekanayake R, Jayawardena R (2014). Prevalence of hospital malnutrition among cardiac patients: results from six nutrition screening tools. Springerplus.

[18] Somasundaram N, Rajaratnam H, Wijeyarathne C, Katulanda P, De Silva S, Wickramasinghe P (2014). Clinical guidelines: The Endocrine Society of Sri Lanka; Management of obesity. Sri Lanka J Diabetes.

[19] Jayawardena R, Byrne NM, Soares MJ, Katulanda P, Hills AP (2014). Body weight perception and weight loss practices among Sri Lankan adults. Obes Res Clin Pract.

[20] Gupta M, Singh N, Verma S (2006). South Asians and cardiovascular risk: what clinicians should know. Circulation.

