# Supplementary material for: Body weight perception among Sri Lankan cardiac patients
Source: BMC Obes. 2016 Jul 4;3:32. doi: 10.1186/s40608-016-0113-5 (PMC4940977; doi:10.1186/s40608-016-0113-5)
Supplement: Additional file 1: — Multiple logistic regression models for under-perception, correct perception and over perception of body weight. (DOCX 17 kb) [file 40608_2016_113_MOESM1_ESM.docx]

**Additional File 01**

After preliminary statistical analysis, multiple logistic regression analysis was carried out for under perception, correct perception and over perception of body weight. Perceived overweight was the dependent variable whereas age, ‘measured’ BMI, ethnicity, gender, education level and the presence of metabolic diseases were identified as independent variables.

BMI was a significant predictor of under perception (OR= 1.23; 95% CI=1.16-1.31), correct perception (OR= 0.86; 95% CI= 0.82-0.91) and over perception (OR= 0.64; 95% CI= 0.96-1.03) of body weight. For over perception of body weight, there was a significant association with male gender (OR= 3.72; 95% CI= 1.18-11.68). None of the other variables had statistically significant associations with perceived weight (p>0.05). Presence or absence of co-morbid metabolic diseases also was not significantly associated with perception of body weight (Table IA).

Due to the lack of clinical relevance in the results, the findings were not included in the study.

Table IA: Multiple logistic regression models for under perception, correct perception and over perception of body weight

| Variables | Under perception | | Correct perception | | Over perception | |
| --- | --- | --- | --- | --- | --- | --- |
|  | OR (95% CI) | p value | OR (95% CI) | p value | OR (95% CI) | p value |
|  |  |  |  |  |  |  |
| Age (years) | 1.00(0.99-1.02) | 0.736 | 0.99(0.98-1.01) | 0.757 | 0.99(0.96-1.03) | 0.736 |
| BMI | 1.23(1.16-1.31) | 0.000 | 0.86(0.82-0.91) | 0.000 | 0.64(0.96-1.03) | 0.000 |
| Education level | 0.86(0.65-1.13) | 0.273 | 1.28(0.97-1.69) | 0.079 | 0.66(0.36-1.21) | 0.184 |
| Female (Ref.) | 1 |  | 1 |  | 1 |  |
| Male | 0.99(0.66-1.51) | 0.992 | 0.89(0.59-1.34) | 0.592 | 3.72(1.18-11.68) | 0.024 |
| Sinhala (Ref.) | 1 |  | 1 |  | 1 |  |
| Others | 1.64(0.93-2.89) | 0.088 | 0.76(0.43-1.33) | 0.339 | 0.22(0.03-1.79) | 0.159 |
| Metabolic diseases present (Ref.) | 1 |  | 1 |  | 1 |  |
| Metabolic diseases absent | 0.68(0.45-1.03) | 0.070 | 1.41(0.93-2.13) | 0.108 | 1.12(0.42-2.97) | 0.818 |
